# Fabrication of a Micro-Needle Array Electrode by Thermal Drawing for Bio-Signals Monitoring

**DOI:** 10.3390/s16060908

**Published:** 2016-06-17

**Authors:** Lei Ren, Qing Jiang, Keyun Chen, Zhipeng Chen, Chengfeng Pan, Lelun Jiang

**Affiliations:** Guangdong Provincial Key Laboratory of Sensor Technology and Biomedical Instrument, Sun Yat-Sen University, Guangzhou 510006, China; rlei@mail2.sysu.edu.cn (L.R.); jqing@mail.sysu.edu.cn (Q.J.); yunzibetterlife@163.com (K.C.); tiaopiji@icloud.com (Z.C.); panchf@mail2.sysu.edu.cn (C.P.)

**Keywords:** micro-needle array, electrode, impedance, EMG, ECG, EEG, finite element method

## Abstract

A novel micro-needle array electrode (MAE) fabricated by thermal drawing and coated with Ti/Au film was proposed for bio-signals monitoring. A simple and effective setup was employed to form glassy-state poly (lactic-co-glycolic acid) (PLGA) into a micro-needle array (MA) by the thermal drawing method. The MA was composed of 6 × 6 micro-needles with an average height of about 500 μm. Electrode-skin interface impedance (EII) was recorded as the insertion force was applied on the MAE. The insertion process of the MAE was also simulated by the finite element method. Results showed that MAE could insert into skin with a relatively low compression force and maintain stable contact impedance between the MAE and skin. Bio-signals, including electromyography (EMG), electrocardiography (ECG), and electroencephalograph (EEG) were also collected. Test results showed that the MAE could record EMG, ECG, and EEG signals with good fidelity in shape and amplitude in comparison with the commercial Ag/AgCl electrodes, which proves that MAE is an alternative electrode for bio-signals monitoring.

## 1. Introduction

Bioelectricity is a typical physiological phenomenon of humans which can provide important information for diagnostics and treatment. However, bioelectric signals are weak and difficult to collect. Electrodes, including wet electrodes (typical Ag/AgCl wet electrode) and dry electrodes, can collect and record bioelectric signals [1]. The micro-needle array electrode, as a dry electrode, has attracted more and more attention for physiological electrical signals monitoring (including EII, ECG, EEG, and EMG recording) in recent decades [2]. In the EII test, the MAE presents less variation of contact impedance and better stability due to the significant improvement of the contact interface between the electrode and skin, compared with conventional Ag/AgCl electrodes and metal planar bio-electrodes [3,4]. In an EMG signals test, both the MAE and Ag/AgCl electrode can trace the change of EMG signals well and easily reduce the crosstalk [2,5]. In static state recording of ECG signals, signals collected by the MAE agree well with that recorded by typical Ag/AgCl electrodes [2,5,6,7,8,9], while in a dynamic state recording of ECG signals, the MAE can capture more obvious ECG principal features due to the reduction of motion artifacts and a stable contact interface between the MAE and human skin [2,7]. In EEG signals measurement, the MAE can pass through the hairs, reach the scalp, and penetrate through the dead skin layer without any obstruction. Sensing performance of the MAE is better in terms of electrode-skin impedance over a long period of recording, as well as a much higher efficiency in preparation of EEG measurements compared with typical Ag/AgCl electrodes [2,10,11]. Furthermore, Ag/AgCl electrodes work well in short term monitoring but, subsequently, the gel dries, causing disruption in the signals [8]. The electrode gel may also cause itching, allergic reactions, and other skin problems in some patients [2,12]. The MAE can be used without gel and can directly pierce through the stratum corneum, lower the impedance between the skin and electrode, reduce motion artifacts, and is convenient for long-term monitoring of bioelectric signals [8,10]. The MAE is a promising alternative to wet electrodes in some specific situations.

Various fabrication methods of the MAE are employed to fabricate MA from silicon, metal, or polymer. Photolithography technology with wet etch or dry etch is always used to fabricate MA on a silicon substrate [5,10,13,14,15]. This method is fit for mass production of MA. However, it has disadvantages: firstly, photolithography or etching needs sophisticated equipment located in a clean room and may produce toxic waste. It is inconvenient, expensive, and eco-unfriendly. Secondly, silicon micro-needles may easily break off and stay in the skin due to its fragile property [5,15]. Thus, metal is adopted to fabricate MA due to its good strength. Nanosecond IR pulsed-laser machining was used to fabricate MA on a pure copper substrate for bio-signals monitoring [3]. Nanosecond pulsed-laser machining has high machining efficiency and flexibility. However, the surface of the micro-needles is usually rough due to the recast layer and debris deposition on it, and the tips are relatively blunt. The biocompatibility of pure copper also needs to be further discussed. Polymer is proposed to fabricate MA due to its good toughness and high yield for the skin insertion of MA. Vacuum-casting technology was employed to fabricate multiple micro-spike electrodes for EEG recording [11]. The master pattern was made by CNC micromachining. This technology can also be applied in mass production while its process is relatively complex. 3D printing technology was also proposed to fabricate 3D MA using biocompatible acrylic-based resin for EEG and ECG recording [6]. This technique allowed for personalized customization and flexibility. However, the resolution of 3D printing was low, the size of MA was in the millimeter scale and the tip diameter was about 100 μm. As the MA was fabricated, a conductive film, such as Ti, Au, Ag, or AgCl, should be subsequently coated on MA by sputtering electroless plating, electrolysis, e-beam evaporation, *etc.* [5,10,13,16,17].

In this paper, we introduce a thermal drawing method to fabricate 3D MA from 2D planar thermosetting polymer film. A simple thermal drawing setup was designed and fabricated. The polymer is heated to a glassy state, drawn into 3D MA by pillar arrays, and finally solidified by thermal curing. Thermal drawing technology is very simple, efficient, and low cost. The technology is also fit for various polymers and mass fabrication. Subsequently, MA is coated with a conductive film of Ti/Au by magnetron sputtering to fabricate the MAE. The biocompatibility of the MAE will be discussed. EII of the MAE and Ag/AgCl electrodes will be measured under compression force. Finite element methods will be proposed to simulate the insertion and pull process of the MAE. The performance of monitoring ECG, EMG, and EEG bio-signals will be evaluated in comparison with commercial Ag/AgCl electrodes.

## 2. Experimental

### 2.1. MAE Fabrication

#### 2.1.1. Experimental Setup

**Figure 1:**
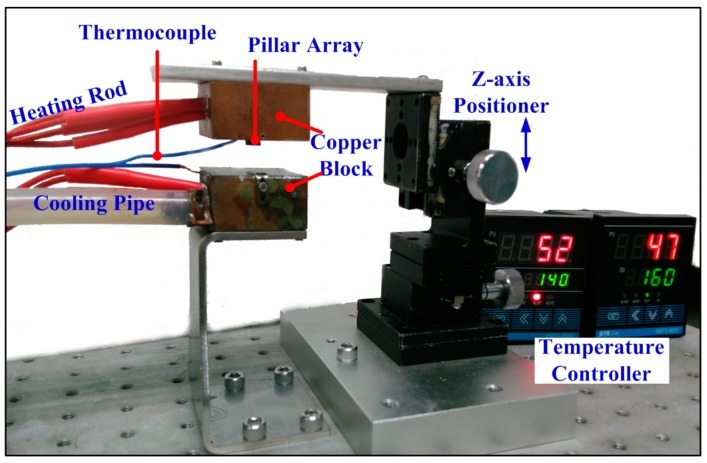
Thermal drawing setup for MA.

A simple and effective experimental setup was designed and fabricated for thermal drawing of MA, as shown in Figure 1. It consists of a temperature adjustment module, *z*-axis positioner, and stainless steel pillar array. In the temperature adjustment module, heating rods are employed to heat copper blocks at a given temperature, and constant temperature water bath pumps low-temperature water to rapidly cool the bottom copper block. The *z*-axis positioner could manually slip along the *z* direction with the pillar array to draw glassy-state polymer into a 3D MA.

#### 2.1.2. Fabrication Process

##### Materials Preparation

Thermosetting polymer, 75/25 PLGA (Mw = 200 kDa) was purchased from Shenzhen Polymtek Biomaterial Co., Ltd. (ShenZhen, China) PLGA has been widely used in tissue scaffolds and is safe for skin penetration [18]. A round PLGA film of 0.6 mm thickness and 10 mm diameter was cut. The material of the pillar array was 316L stainless steel, the height was 3 mm, and its diameter was 350 μm. The pillars were welded on the upper copper block in an array of 6 × 6 with a separation of 1 mm.

##### Thermal Drawing of MA

**Figure 2:**
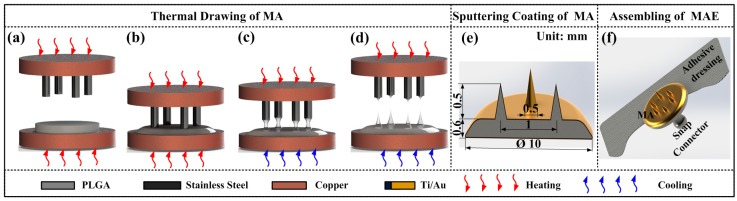
Fabrication process of MAE. (**a**)–(**d**): thermal drawing process of MA, (**e**) Sputtering coating Ti/Au film on the MA, and (**f**) the assembly of MAE.

The thermal drawing process of MA is shown in Figure 2a–d. Firstly, the round PLGA film was fixed to the bottom copper block and heated at 140 °C. The PLGA film was heated into a glassy state, as shown in Figure 2a. The upper copper block was heated at 160 °C to preheat the pillar array. Secondly, the pillar array was moved down towards the glassy-state PLGA film until the pillar array touched the PLGA film, as shown in Figure 2b. Thirdly, the pillar array was moved upward at a speed of 0.25 mm/s and then necks were formed between the pillar array and PLGA film due to the surface tension and became narrower. Fourthly, the bottom copper block was rapidly cooled to room temperature (about 25 °C), as shown in Figure 2c. Finally, the pillar arrays were move upward continuously and the necks were broken due to the high temperature of the pillar array. MA tips were formed, as shown in Figure 2d.

##### MA Coating

10 nm Ti film and 100 nm Au film were uniformly coated on the whole MA surface in sequence by a magnetron sputtering machine (MSP-3300, Beijing Jinshengweina Technology Co., Ltd, Beijing, China) as shown in Figure 2e. The coating Ti film was used to increase the bonding strength between the PLGA and Au film [6]. The coating Au film could insure the conductivity of the MAE [6,14]. The coated micro-needle array was observed by scanning electron microscopy (SEM, JSM-6380LA, JEOL, Tokyo, Japan).

##### MAE Assembling

An electrode button was glued to the backside of the MA by conductive silver glue and a medical adhesive dressing was bonded with a standard-shape snap connector, as shown in Figure 2f. The medical adhesive dressing could insure MAE sticks on, and penetrates into, the skin. The standard snap connector could guarantee the connection between the MAE and common bio-signals recording devices.

### 2.2. Insertion, EII Test, and Numerical Simulation

#### 2.2.1. EII Test during the Insertion Process

**Figure 3:**
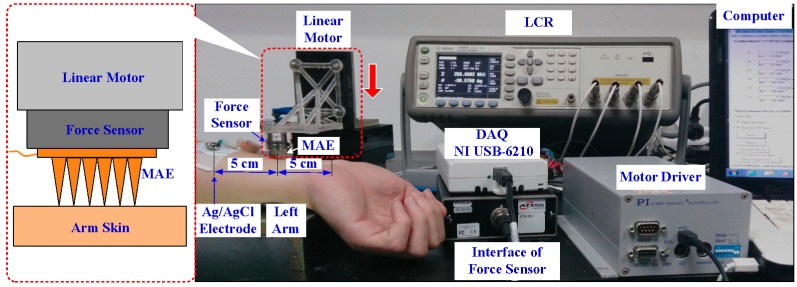
Setup for EII recording during the insertion process.

The MAE can penetrate through the stratum corneum layer and eliminate the impendence of the dead skin to collect EII. We designed a setup to test the EII of the MAE during the insertion process, as shown in Figure 3. The left inner forearm was chosen as the measurement object due to having less hair, a thinner stratum corneum, as well as being a convenient and accurate position for electrodes [3,19]. A two-electrode measurement method was proposed to record EII [3]. The location of the MAE and Ag/AgCl electrode is shown in Figure 3. The electrodes were connected to the precision impedance analyzer (Agilent E4980A LCR Meter, Palo Alto, CA, USA) with coaxial wires, to avoid noise. A linear motor (E-861, PI, Karlsruhe, Baden-Württemberg, German) could load the MAE on the forearm and the force sensor (Nano 17 Titanium, ATI Industrial Automation, Detroit, MI, USA) captured the insertion force. The EII signal, insertion force, and displacement could be simultaneously recorded by self-developed software during the insertion process.

In order to better understand the effect of insertion force on the recording of EII, the EII of the MAE and insertion force were recorded during the insertion process. The linear motor speed was 0.5 mm/s, the injection voltage of the LCR meter was set at 1 V, and its frequency was set at 50 Hz. We also recorded EII with the frequency from 20 Hz to 10 kHz when the insertion force was held at a constant value (about 1 N, 2 N, or 3 N). As the measured impedance is beyond 2 MΩ, we set the test result as 2 MΩ.

#### 2.2.2. Numerical Simulation of Insertion and Pull Process

**Figure 4:**
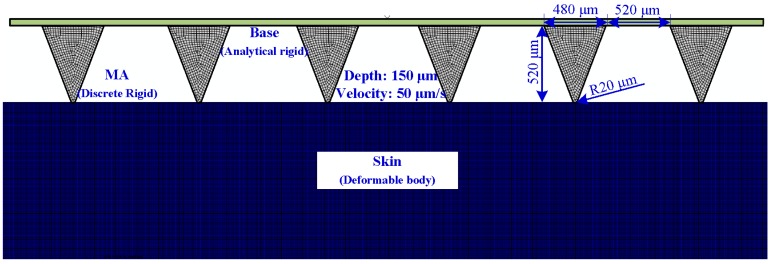
Finite element model of the MAE insertion and pull process.

The mechanism of the MAE insertion and pull process to human skin has not yet been understood clearly. We proposed a nonlinear FEM model to simulate this process by ABAQUS Explicit as shown in Figure 4. For simplicity, we simulated it with a 2-D plane-strain model under quasi-static conditions. The MAE was inserted into the skin at a vertical direction and drawn from the skin at a speed of 50 μm/s. The insertion depth was 150 μm.

Human skin consists of four layers: stratum corneum, epidermis, dermis, and hypodermis. We assumed the skin of each layer as the isotropous and incompressible material to simplify the simulation. The Neo-Hooken constitutive model of skin was employed [20,21] and the mechanical properties are listed in Table 1 [22,23,24]. The hypodermis layer was ignored in the model since the MAE could not reach and its effect was minimal. The material model of skin was defined by the VUMAT subroutine in ABAQUS. The failure criterion handled by the distortion energy theory was introduced in the skin material model. When the effective stress of an element near the micro-needle tips was beyond the specified failure criterion, it would be identified and eliminated from the mesh. The skin was defined as a deformable body and meshed with 12,000 CPE4R elements, each micro-needle was defined as a discrete rigid body and meshed with 469 CPS4R elements, and the base of the MAE was defined as an analytical rigid body.

Surface-to-surface contact was defined between micro-needles and skin using the kinematic contact algorithm. Contact between the MAE and skin was modeled with the friction coefficient of 0.42 [25]. The rotation of the X, and X and Y translation were constrained in the nodes of the MAE. The displacement boundary conditions on the skin included nodes along the bottom, left, and right edges that were pinned, whereas the top edge was traction-free. The explicit method was introduced to calculate the process.

### 2.3. Bio-Signals Recording

In order to better understand the sensing performance of MAE, bio-signals, including EMG, ECG, and EEG, were recorded by the MAE in comparison with conventional Ag/AgCl electrodes (JK-1(A~H) type, Shanghai Junkang Medical Supplies LTD., CO, Shanghai, China). Measurements were firstly performed with the MAE and then repeated with Ag/AgCl electrodes under the same test conditions. The electrodes were located at the same position and followed the same procedures in both cases. The experiments were operated on three healthy volunteers from 23 to 26 years old at room temperature and repeated at least five times.

#### 2.3.1. EMG Test

**Figure 5:**
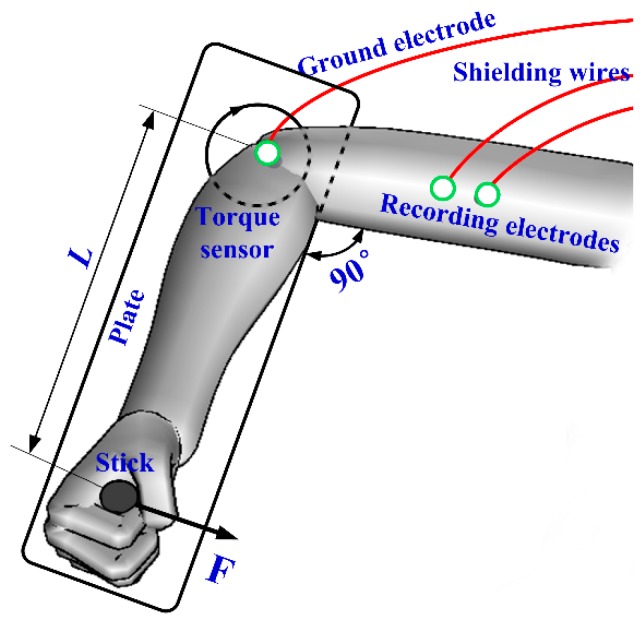
Schematic diagram of EMG measurement.

The biceps brachii muscle of the right arm was employed as the EMG measuring object, as shown in Figure 5. The differential method was introduced to record EMG signals. The upper arm was placed on the plate and the right hand held the stick. The stick was assembled on one end of plate. The angle between upper arm and forearm was 90°. If the upper arm tried to rotate the plate, the torque sensor (AKC-205, 701st Research Institute of China Aerospace Science and Technology Corporation, Beijing, China) are the other end of the plate would record the torque value. Two recording electrodes were stuck on the biceps brachii with a distance of 2 cm and a ground electrode was placed on the elbow. These electrodes were connected to a tele-EMG system (MyoSystem2400T, Noraxon, Scottsdale, AZ, USA). EMG signals were collected by data acquisition card (DAQ-6341, National Instruments, Austin, TX, USA) and analyzed by a customized LabVIEW program (LabVIEW 2012, National Instruments Corporation, Austin, TX, USA). The skin was firstly cleaned with medical alcohol. The volunteer held the stick and increased the torque from 0 Nm to 15 Nm. As the torque reached about 15 Nm, the volunteer held for about 5 s. Then, the volunteer released the force. The torque and EMG signals were recorded simultaneously. The process was be repeated several times.

#### 2.3.2. ECG Test

The MAE sensing performance of ECG signals was operated in static and dynamic state by standard II-lead method. Electrodes were connected to an ECG100C module from Multipurpose Polygraph (MP150, BIOPAC, Goleta, CA, USA). Measuring electrodes were stuck on the right wrists and left ankle, and grounded electrode was on the right ankle. The volunteer lay in a bed during the static state test and walked on a treadmill at a uniform velocity of 3 km/h during the dynamic state test. The test lasted about half an hour. 

#### 2.3.3. EEG Test

EEG measures voltage fluctuations resulting from ionic current within the neurons of the brain. A unipolar connection method was used in the EEG test. Three electrodes were connected to the EEG100C module of Multipurpose Polygraph, two measuring electrodes and one ground electrode. One measuring electrode was placed on the standard position (Fp1) of the 10–20 system. The ground electrode and the other measuring electrode were located on the left earlobe. The volunteers were asked to blink for 30 s and rest for 30 s during the blinking test. Volunteers then were required to alternate closed and opened eyes with an interval of two seconds under the tester’s guide during the eyes closed and open transition test. The whole test process last about 5 min.

## 3. Results and Discussion

### 3.1. Characterization of the MAE

**Figure 6:**
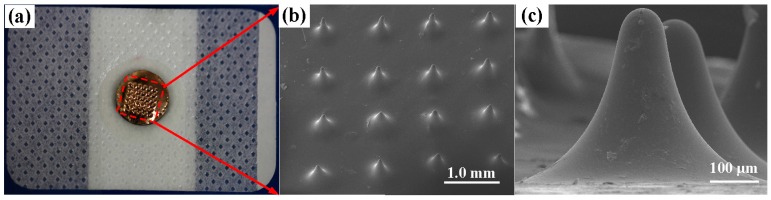
(**a**) Photo of the MAE; (**b**) SEM image of MA; and (**c**) SEM image of the micro-needles.

The MAE fabricated by thermal drawing and coated with Ti/Au film is shown in Figure 6a. The MAE mainly consists of PLGA MA and its Ti/Au film. PLGA, Ti, and Au are all biocompatible which can guarantee the compatibility of the MAE in human skin [18,26,27,28,29]. The MA consists of 6 × 6 micro-needles with an interval of 1 mm, which matches well with that of the stainless steel pillar array. The height of MA distributes uniformly as shown in Figure 6b. The average height is 500 ± 10 μm, which is appropriate for painless recording [10]. The MA surface was very smooth, which may decrease the sliding friction force between skin and MA during the penetration. One micro-needle of MA is shown in Figure 6c. The shape of the micro-needle looks like a cone. The base diameter of micro-needles was about 500 ± 10 μm and its radius of tip curvature was about 40 ± 2 μm. The base diameter was kept large to strengthen the micro-needle and avoid buckling during the skin insertion.

### 3.2. EII Test and Insertion Process

#### 3.2.1. EII Recording during MAE Compression

**Figure 7:**
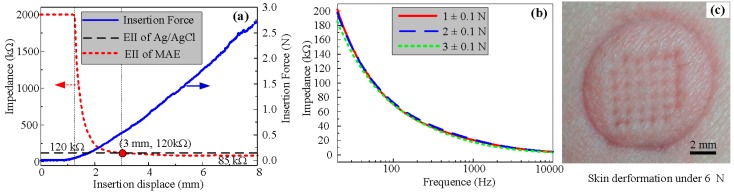
Insertion force and EII test. (**a**) EII during the insertion process, (**b**) EII under different input current frequency, and (**c**) skin deformation under the impression force of 6N.

The EII of MAE during the insertion process at 50 Hz is shown in Figure 7a. The EII value is extremely high at the beginning and the compression force is null due to the absence of contact between the MAE and skin. Subsequently, micro-needle tips come in contact with forearm skin and the insertion force gradually increases, and EII maintains its high impedance value due to the high impedance of the stratum corneum layer. As the compression force is about 75 mN, EII suddenly changes the slope. It may be due to the penetration of one micro-needle through the stratum corneum and the contact impedance between the MAE and skin decreases rapidly. As the MAE is moved forward, the micro-needles of the MAE penetrates into the skin one by one and the EII decreases with the insertion depth. When a force larger than 0.55 N is applied on the MAE, the MAE achieves lower EII than the Ag/AgCl electrodes (120 KΩ). When the compression force on the MAE is larger than 1 N, EII of the MAE reaches a steady state, and its value is about 85 KΩ. The average force of an adult applying the MAE with their thumb is about 20 N [30]. Therefore, we can easily press the MAE into skin with a thumb and stably record the bio-signals.

The MAE impedances of skin-electrode under different compression forces at the driving current frequency from 20 Hz to 10 kHz are shown in Figure 7b. The EII of MAE decreases with the frequency. The impendence of the human body always consist of resistance and capacitive resistance. The capacitive resistance decreases with the frequency of inject driving current. The EII slightly decreases with the insertion force at a given driving current frequency, as shown in Figure 7b. The measured EII usually consists of contact impedance, electrode impedance, and tissue impedance. The contact impedance between the electrode and skin slightly decreased with the insertion force since both the electrode impedance and tissue impedance maintain a constant value at a given current frequency. Therefore, the MAE can record EII or bio-signals at a relatively low insertion force. As the compression force on the MAE is beyond about 5 N, the subjects would feel tingling during the insertion process. Since the MAE can stably record EII under a compression force of 1 N, the bio-signals recording on the subjects is almost painless. The skin deformation under the insertion force of 6 N is shown in Figure 7c. The mark on the forearm skin by the MAE will gradually disappear in about 8 min and the harm on skin is minimal.

#### 3.2.2. Numerical Simulation of the Insertion and Pull Process

**Figure 8:**
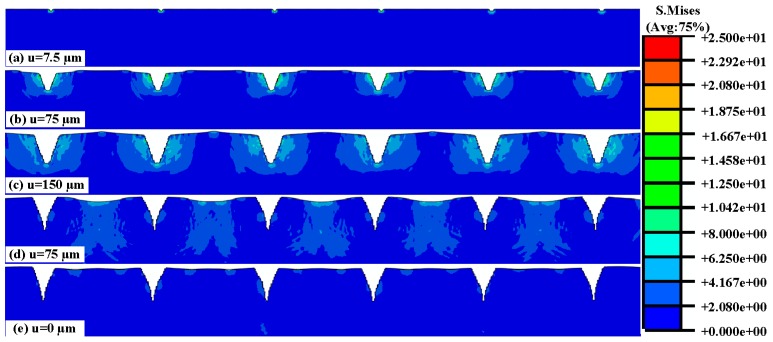
Stress distribution on human skin during the insertion and pull process.

Figure 8a–c present the skin stress distribution of the insertion process. As the micro-needle tips touch the top layer of skin, the stress concentration occurs at the tip area of MAE as shown in Figure 8a. Top layer skin initially deforms concave downward under the advancing tips until a critical stress leads to the skin penetration. Once the effective stress of skin elements near the tips exceeds the specified failure criterion, the nodes are separated due to the deletion of “dead” elements. Thus, the micro-needle tips “tear” the human skin. The skin stress concentration is always on the micro-needle tips during the insertion process, which verifies that the skin tissue is cut away by the MAE tips, as shown in Figure 8a–c. The stress concentration at the micro-needle surface may lead to the increase of the friction force between micro-needles and skin. The friction force increases with the contact area between micro-needles and skin as the MAE inserts in the skin more deeply. It fits well with the plot of insertion force *vs.* displacement is shown in Figure 7a. The stress interaction of adjacent micro-needles can be ignored during the insertion process as shown in Figure 8a–c, so the distance of adjacent micro-needles (1 mm) is suitable for MAE penetration. Figure 8c–e present the stress distribution of the pull process. As the MAE is drawn back, the compression state of the skin near the micro-needles turns into a tensile state. The profile of skin deforms from concave to convex, and finally becomes flat. The taper holes gradually shrink due to the elasticity of skin during the pull process. The shape of insertion holes fits with the skin deformation in Figure 7c.

### 3.3. Bio-Signals Measurement

#### 3.3.1. EMG Measurement

**Figure 9:**
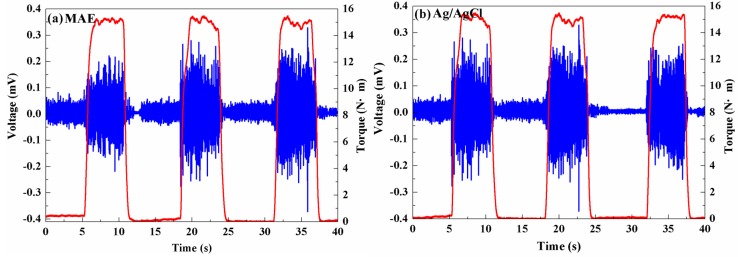
EMG signals recorded by: (**a**) MAE; and (**b**) Ag/AgCl electrodes.

EMG signal has a variety of biomedical applications, such as a diagnostics tool for identifying neuromuscular diseases, a research tool for studying kinesiology, a control signal for prosthetic devices, and so on. Surface EMG signals of the biceps brachii muscle recorded by the MAE is shown in Figure 9a as the right arm applied force to rotate the stick. As the torque is about 0 Nm, the electrical potential of biceps brachii muscle cells is in a low amplitude. When the torque increases from 0 Nm to 15 Nm, the muscle cells are electrically or neurologically activated, and the electrical potential increases rapidly to a high amplitude. The biceps brachii muscle is contracted from the relaxation state. When the volunteer holds the stick with the torque of 15 Nm for 5 s, the EMG signal maintains a high value due to the sustained contraction of the muscle. The biceps brachii muscle is released and the EMG signal is at a low amplitude again as the rotation force is unloaded. The EMG recording process by the MAE suggests that the MAE can trace the change of EMG signals. The EMG signal of the biceps brachii muscle recorded by the conventional Ag/AgCl electrode also shows a similar profile in comparison with the MAE presented in Figure 9. It demonstrates that the MAE can clearly sense and record the surface EMG signal with good fidelity. The contact surface of the conventional Ag/AgCl electrode is about 200 mm^2^ and the MAE is only about 78.5 mm^2^. The MAE is more suitable for use on the small muscles. Furthermore, micro-needles distribution, number, and size of the MAE can be designed and customized by thermal drawing method according to the shape and size of muscle monitored. Thus, the MAE may be more convenient for both improvement of EMG signal selectivity and elimination of the crosstalk. This is a critical phenomenon in the case of several muscles present in a small space or when the volunteers have small anthropometric dimensions. Therefore, the MAE is also a good choice in EMG recording for potential medical applications due to its good sensitivity and fidelity.

#### 3.3.2. ECG Measurement

**Figure 10:**
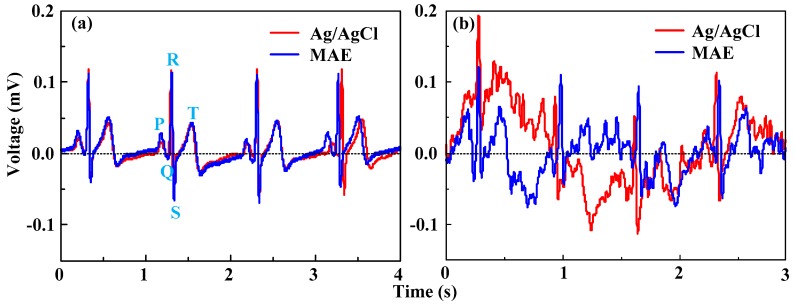
ECG signals recorded: (**a**) in the static state, and (**b**) in the dynamic state.

An ECG signal is always used to test the frequency rate of heartbeats, the size and position of the heart chambers, the presence of any damage to the heart's muscle cells, the effects of cardiac drugs, and the function of implanted pacemakers [31]. Figure 10a presents 4-s ECG recording results using the MAE and Ag/AgCl electrodes from the static state test. The recording performance of the MAE is comparable with Ag/AgCl electrodes. The features of the ECG signal, such as the QRS complex, T and P waves, are all distinguishable as reported in [2,7,32]. The heart rate in this subject is about 65 beats per minute. Therefore, the MAE can record the characteristic ECG peaks effectively in the static state. Figure 10b presents 3-s ECG recording results using the MAE and Ag/AgCl electrodes from the dynamic state test. The ECG signals are seriously affected by motion artifacts [7,33]. As the subject swung the arms during walking on the treadmill, the skin shifted in respect of underlying tissues, modifying the electrodes relative positions and generating the signal drifts and motion artifacts. The signals measured by both the Ag/AgCl electrodes and the MAE are seriously disturbed by noise due to the motion artifacts in comparison with the signals recorded in the static state. The typical R waves and T waves of the MAE results are recognizable and the heart frequency rate can be calculated. ECG signal collected by the MAE has more distinguishable QRS complex, P waves and T waves than signal by Ag/AgCl electrodes. The ECG signals collected by Ag/AgCl electrodes seriously shift from the baseline. We infer that the MAE can insert into the skin and maintain a relatively more stable electrode-skin contact interface.

**Figure 11:**
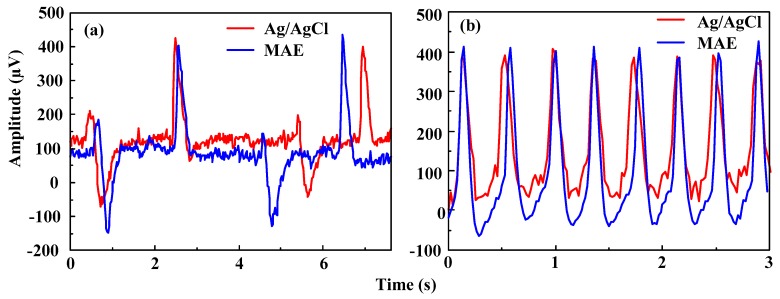
EEG signals recorded by Ag/AgCl electrodes and the MAE: (**a**) closed and opened eyes transition, and (**b**) blinking the eyes.

EEG signals can be used to diagnose epilepsy, sleep disorders, coma, encephalopathies, brain death, and so on. The EEG is more difficult to capture since its amplitude is in the order of μV while the ECG is in the range of mV. EEG signals recorded on Fp1 by MAE and Ag/AgCl electrodes are shown in Figure 11. Signal profiles captured by the MAE are very similar with that of the Ag/AgCl electrodes during the eyes closed and eyes open transition as shown in Figure 11a. Both beta rhythm (eyes open condition) and alpha rhythm (eyes closed condition) are recognizable, as found in [10]. When the subject was asked to blink the eyes successively, the blink signal comes in on the EEG recording and fluctuates regularly. The same fluctuations are observed by both the MAE and Ag/AgCl electrodes, as shown in Figure 11b. Both the MAE and Ag/AgCl electrodes can monitor EEG signals, but MAE could directly capture bio-signals without skin preparation (such as hair cutting and skin abrasion) and application of electrolytic gel. It may be more comfortable for subjects without skin abrasion, skin allergy, and the bother of the gel drying in long term monitoring [2].

## 4. Conclusions

Micro-needle array electrode was developed for bio-signals monitoring in this paper. The insertion process of the MAE in the skin was simulated, and bio-signals recording performance, including EII, EMG, ECG, and EEG, were investigated by experiments. We obtained the main conclusions:

(1) The MAE, biocompatible for humans, can be fabricated by a thermal drawing method and magnetron sputtering in mass production. The micro-needle tips can “tear” and insert the human skin due to the stress concentration near the tips. When the compression force on the MAE is larger than 1 N, EII of the MAE is about 85 KΩ which is lower than that measured by Ag/AgCl electrodes (120 KΩ). EII slightly decreases with the insertion force at a given driving current frequency. The MAE can record EII at a relatively low insertion force.
(2) The MAE can clearly sense and record the surface EMG signals as well as Ag/AgCl electrodes. The geometry of the MAE can be customized by a thermal drawing method based on a monitored muscle to improve the selectivity of EMG and eliminate the crosstalk. The ECG features measured by both the MAE and Ag/AgCl electrodes are all distinguishable in the static state. ECG signals in the dynamic state measured by both Ag/AgCl electrodes and the MAE are seriously disturbed by motion artifacts, but ECG signals of the MAE is more distinguishable than the signal collected by Ag/AgCl electrodes. Both the MAE and Ag/AgCl electrodes can record EEG signals. The MAE could directly capture bio-signals without skin preparation and application of electrolytic gel.

MAE seems to be a promising alternative electrode to conventional Ag/AgCl electrodes in bio-signal monitoring. The comfort of MAE for patients during the long-term acquisitions and the strength of the micro-needles need to be further improved. Additionally, before the proposed MAE electrode is applied for EEG monitoring in hairy positions, a special fixture with a suitable pressure on the head should be designed and fabricated.

## Figures and Tables

**Table 1:** Mechanical properties of human skin.

	Stratum Corneum	Epidermis	Dermis
C_10_ (MPa)	10	0.11	0.16
Failure stress (MPa)	25	--	7.3
Density (kg/m^3^)	1300	1200	1200
Thickness (mm)	0.07	0.05	0.1
